# The Role of Mondo Family Transcription Factors in Nutrient-Sensing and Obesity

**DOI:** 10.3389/fendo.2021.653972

**Published:** 2021-03-31

**Authors:** Huiyi Ke, Yu Luan, Siming Wu, Yemin Zhu, Xuemei Tong

**Affiliations:** Department of Biochemistry and Molecular Cell Biology, Shanghai Key Laboratory for Tumor Microenvironment and Inflammation, Key Laboratory of Cell Differentiation and Apoptosis of Chinese Ministry of Education, Shanghai Jiao Tong University School of Medicine, Shanghai, China

**Keywords:** MondoA, ChREBP, nutrient-sensing, obesity, brown and beige adipose tissue

## Abstract

In the past several decades obesity has become one of the greatest health burdens worldwide. Diet high in fats and fructose is one of the main causes for the prevalence of metabolic disorders including obesity. Promoting brown or beige adipocyte development and activity is regarded as a potential treatment of obesity. Mondo family transcription factors including MondoA and carbohydrate response element binding protein (ChREBP) are critical for nutrient-sensing in multiple metabolic organs including the skeletal muscle, liver, adipose tissue and pancreas. Under normal nutrient conditions, MondoA and ChREBP contribute to maintaining metabolic homeostasis. When nutrient is overloaded, Mondo family transcription factors directly regulate glucose and lipid metabolism in brown and beige adipocytes or modulate the crosstalk between metabolic organs. In this review, we aim to provide an overview of recent advances in the understanding of MondoA and ChREBP in sensing nutrients and regulating obesity or related pathological conditions.

## Introduction

The epidemic of obesity has emerged as a worldwide public health issue. In 2017, the Global Burden of Disease Study estimated that high body mass index (BMI), one of the leading risk factors, accounted for 4.72 million deaths and 148 million disability-adjusted life-years (DALYs) (1). Excessive caloric intake mainly derived from the high-fat and high-fructose diet is a major cause for obesity (2–4). The urgent need for weight-loss treatments has given rise to multiple attempts to target cellular metabolism and restore systemic energy homeostasis, among which is the strategy of promoting brown and beige adipocyte activity and development. Brown adipocytes are essential for thermogenesis in mammals with their characteristic expression of uncoupling protein-1 (UCP1) in mitochondria, while beige adipocytes are inducible to express thermogenic genes in response to stimulus (5). Enhancing activities of brown and beige adipocytes not only promotes energy expenditure through heat generation, but also enhances glucose metabolism and protects against insulin resistance (6–11), which provides promising therapeutic effects to counteract obesity and related diseases.

**Figure 1:**
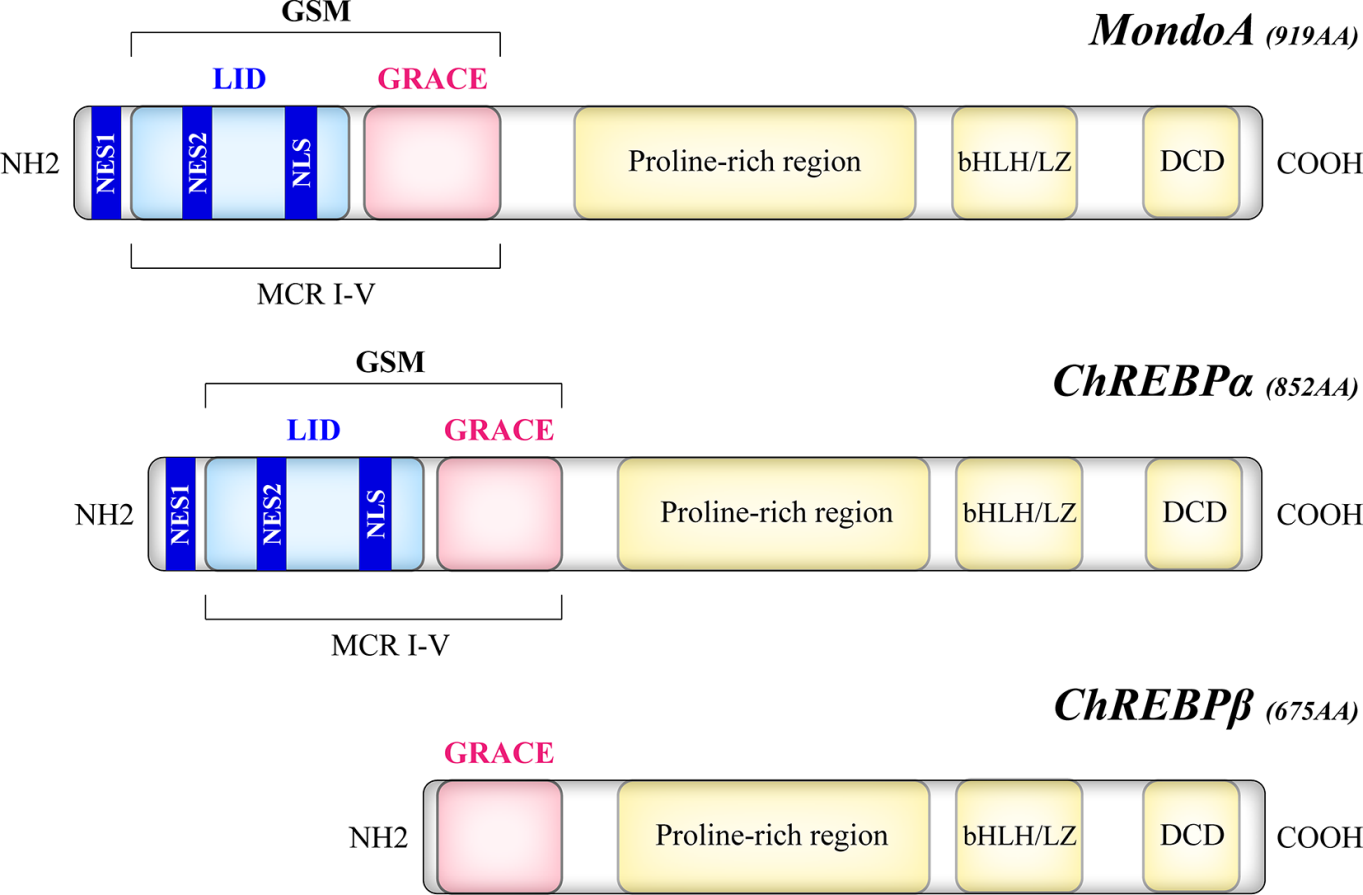
Structural domains of the human Mondo proteins. MondoA and ChREBP contain homologous C-termini, where a bHLH/LZ region and a dimerization and cytoplasmic localization domain (DCD) mediate the heterodimerization process and DNA binding. On the other hand, the N-termini of MondoA and ChREBP determine their glucose responsiveness. The glucose-sensing module (GSM) lies within the N-terminal region of MondoA and ChREBPα and is composed of a low-glucose inhibitory domain (LID) and a glucose-response activation conserved element (GRACE). Compared with the canonical ChREBPα of 852 amino acids, the ChREBPβ isoform, a product of alternative splicing, is a 675-amino acid protein that lacks the LID domain. The N-terminal region also includes five Mondo conserved regions (MCR I-V), with LID spanning MCR I-IV and GRACE harboring MCR V. Two nuclear export signals (NES1, NES2) correspond to the binding site of CRM1, while a nuclear localization signal (NLS) allows the interaction with importin-α.

The Mondo family transcription factors, comprised of MondoA (also known as MLXIP) and carbohydrate response element binding protein (ChREBP, also named MondoB and MLXIPL), belong to the basic helix-loop-helix leucine zipper (bHLH/LZ) family (12, 13). Upon binding to their heteromeric partner MLX (Max-like protein X), Mondo and MLX translocate to the nucleus where they bind to carbohydrate response elements (ChoREs) on target gene promoters, and stimulate a transcriptional response to nutrients (12–14). As a structural basis of their nutrient-sensing and responsiveness, the glucose-sensing module (GSM) of Mondo proteins consists of a low-glucose inhibitory domain (LID) and a glucose-response activation conserved element (GRACE) (**Figure 1**). Under basal conditions, GRACE is repressed by the LID domain, which is relieved by alterations in nutrient levels such as the elevation of glucose concentration (15). An isoform of ChREBP, ChREBPβ, lacks the LID domain and is induced by the activation of the canonical isoform ChREBPα (16). Upon activation, MondoA and ChREBP bind to importin-α which mediates their nuclear entry (17), while their nuclear export and cytoplasmic retention are regulated by chromosome region maintenance protein 1 (CRM1) and 14–3-3 proteins (18–20). Though similar in structure, MondoA and ChREBP have distinct tissue distribution patterns, with MondoA predominantly in skeletal muscle and immune cells and ChREBP in liver and adipose tissue (12, 13), and our unpublished data.

Initially identified as glucose sensors, Mondo family has more extensive regulatory functions in metabolic homeostasis. Therefore, their role in physiological and pathological conditions has gained growing interest. In this review, we will discuss how MondoA and ChREBP sense and respond to nutrient availability, focusing on the involvement of Mondo family in obesity and related diseases.

## Nutrient-Sensing by MondoA and ChREBP

### ChREBP in Metabolic Organs

ChREBP is widely expressed in metabolic organs, predominantly in liver, also in adipose tissues, pancreas, intestine, kidney, relatively low in skeletal muscle (21).

**Figure 2:**
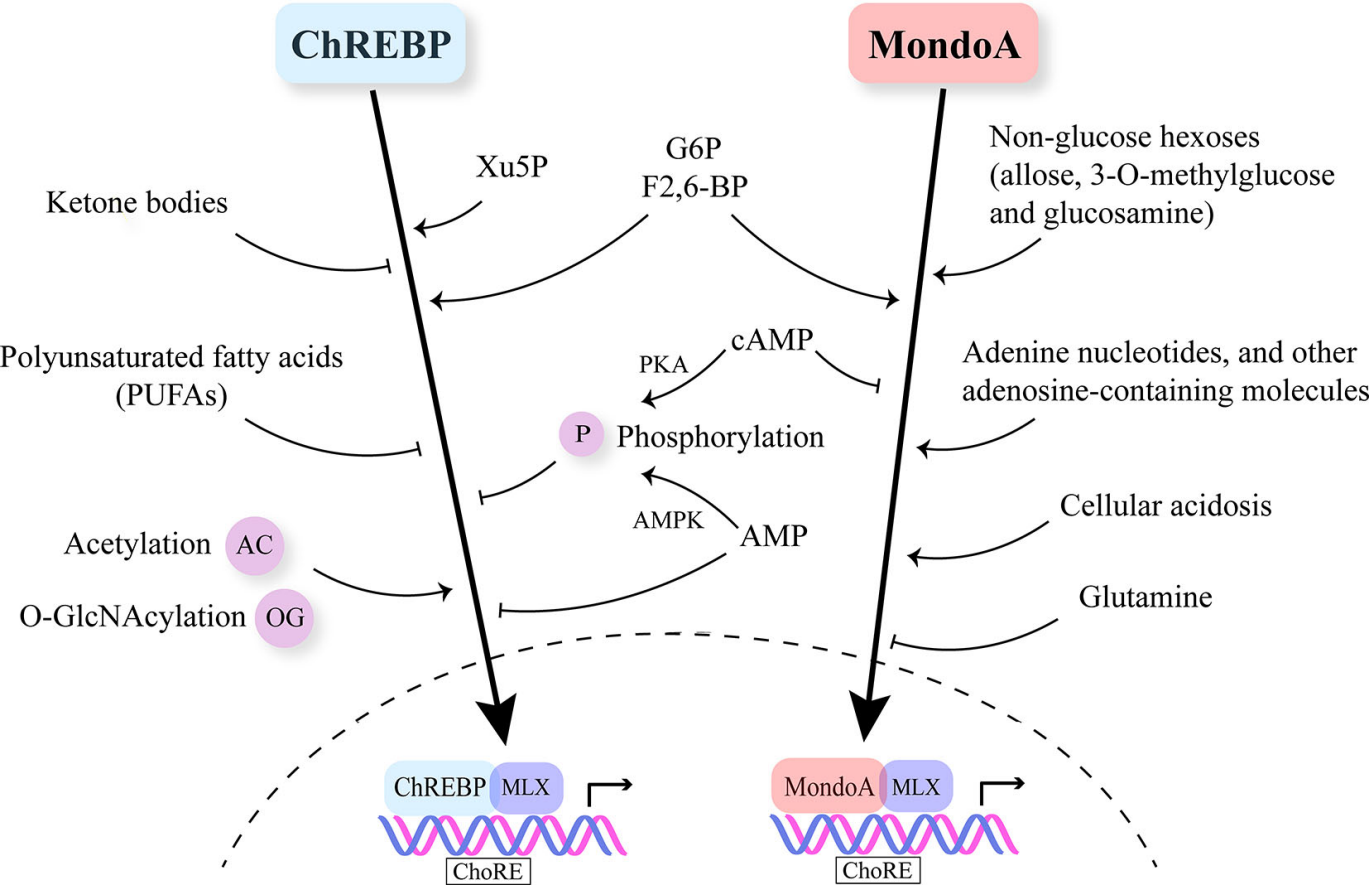
Nutrient-sensing and regulation of Mondo family. Mondo family transcription factors sense multiple nutrients. G6P, F2,6-BP and Xu5P are considered the major metabolites through which glucose stimulates ChREBP and MondoA nuclear translocation and transcriptional activity. Ketone bodies and polyunsaturated fatty acids (PUFAs) are reported to inhibit ChREBP activity. MondoA activators include non-glucose hexoses, adenosine-containing molecules and cellular acidosis. Glutamine represses MondoA transcriptional activity. Post-transcriptional modifications (PTMs) including phosphorylation, acetylation and O-GlcNAcylation also play a role in regulating Mondo family, especially ChREBP. cAMP is a common inhibitor of ChREBP and MondoA. cAMP acts through PKA (protein kinase A) to promote the phosphorylation of ChREBP. Increased AMP levels lead to both retention of ChREBP in the cytosol and AMPK-induced phosphorylation of ChREBP, thus inhibiting ChREBP activity.

ChREBP is regulated by multiple nutrient molecules, among which glucose and its metabolites are major determinants. In the presence of high glucose, glucose 6-phosphate (G6P), the first intermediate in glycolysis binds to the GRACE domain of ChREBP (22, 23). Moreover, xylulose 5-phosphate (Xu5P), the metabolite generated through the pentose phosphate pathway, activates protein phosphatase 2A (PP2A), and the sequential dephosphorylation of several residues activates ChREBP (24). Furthermore, fructose-2,6-bisphosphate (F2,6-BP) derived from fructose-6-phosphate (F6P) has been identified as another signaling metabolite responsible for glucose-induced recruitment of ChREBP to its target genes (25). On the other hand, when glucose is limited, branched chain keto-amino acids (BCKA) and fatty acids (FA) inhibit ChREBP activity (26, 27) (**Figure 2**).

**Figure 3:**
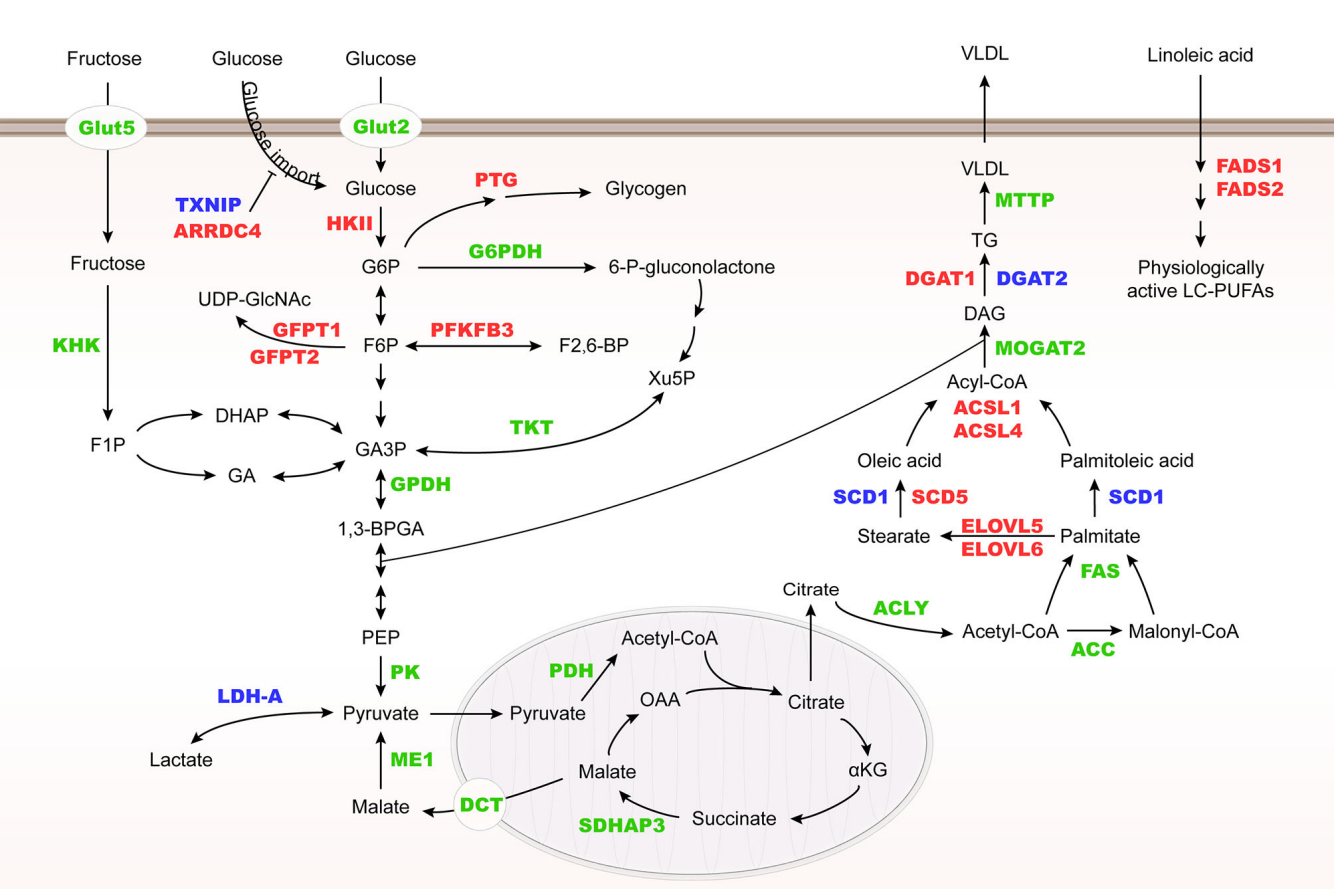
Metabolic genes regulated by Mondo family. Metabolic genes regulated by MondoA and ChREBP at the transcriptional level are herein summarized. They are involved in glucose and fructose uptake, glycolysis, fructose metabolism, glycogenesis, hexosamine biosynthesis pathway (HBP), pentose phosphate pathway (PPP), and lipogenesis. MondoA targets are highlighted in red, ChREBP targets in green, and their common targets are in blue. HK II, hexokinase II; PTG, glycogen targeting protein; G6PDH, glucose-6-phosphate dehydrogenase; KHK, fructokinase; GFPT, glutamine:fructose-6-phosphate aminotransferase; PFKFB3, 6-phosphofructo-2-kinase/fructose-2,6-biphosphatase 3; TKT, transketolase; PK, pyruvate kinase; PDH, pyruvate dehydrogenase; SDHAP3, succinate dehydrogenase complex flavoprotein subunit A pseudogene 3; DCT, C4-dicarboxylate transport protein; ME1, malic enzyme; MOGAT2, monoacylglycerol O-acyltransferase 2; DGAT, diacylglycerol acyltransferase.

ChREBP regulates many enzyme genes in glycolysis and lipogenesis, including liver type pyruvate kinase (L-PK), acetyl-CoA carboxylase (ACC), fatty acid synthase (FAS), ATP-citrate lyase (ACLY), stearoyl-CoA desaturase-1 (SCD1) and glycerol-3-phosphate dehydrogenase (GPDH) (28–30). In addition, ChREBP may control very low-density lipoprotein (VLDL) export by regulating microsomal triglyceride transfer protein (MTTP) transcription (31) (**Figure 3**).

Mouse models with knockout or overexpression of the ChREBP gene provide direct evidence for its role in glucose and lipid metabolism (**Table 1**). ChREBP global knockout mice show down-regulated pyruvate production and inhibited glycolysis, with lower mRNA levels of ACC, FAS, ACLY and SCD1 in liver than wild-type mice, leading to a significant decrease in lipids converted from glucose (32). ChREBP liver-specific knockout mice showed dysregulation of glucose response and impaired glucose homeostasis (34). Moreover, adenoviral overexpression of ChREBP caused higher liver triglyceride contents with increased FAS and ACC expression (35, 46). It is now believed that ChREBP and sterol regulatory element binding protein-1c (SREBP-1c) play a synergistic role in the regulation of lipogenesis in liver (47). Moreover, the expression of the ChREBPβ isoform is associated with the respective increase and repression of branched chain alpha-keto acid dehydrogenase kinase (BDK) and protein phosphatase Mg^2+^/Mn^2+^-dependent 1K (PPM1K) transcript levels in liver (48). In addition, the transcription of fibroblast growth factor 21 (FGF21) is regulated by ChREBP (49). FGF21 is involved in energy metabolism by regulating carbohydrate intake (50). Fructose ingestion increases FGF21 production in a ChREBP-dependent manner while FGF21 knockout attenuates ChREBP expression and *de novo* lipogenesis following fructose consumption, indicating that ChREBP and FGF21 constitute a signaling axis which mediates an adaptive hepatic metabolic response to fructose ingestion (51).

**Table 1:** Roles of Mondo family on body weight, hepatic steatosis and insulin sensitivity according to mouse models, depending on nutritional status, genetic background and drug administration.

Mondo family	Context	Modulation in Mouse Models	Body Weight	Fat Mass	Hepatic Steatosis	Insulin Sensitivity	Reference
ChREBP	Standard diet	Global knockout	=	↘	=	↘	(32, 33)
		Liver-specific knockout	=	↘	=	↘	(34)
		Liver-specific overexpression	=	↘	↗	=	(35)
		AT-specific knockout	=	=	↘	↘	(36)
		AT-specific overexpression	↘	↘	=	=	(37)
		Pancreatic β-cell-specific overexpression	↘	ND	ND	↘	(38)
	Standard diet in *ob/ob* mice background	Global knockout	↘	↘	↘	↗	(33)
		Liver-specific knockdown	↘	↘	↘	↗	(39)
	High-fat diet	Liver-specific knockout	=	=	=	↘	(34)
		Liver-specific overexpression	=	↘	↗	↗	(35)
		AT-specific knockout	=	=	=	↘	(36)
		AT-specific overexpression	↘	↘	↘	↗	(37)
	Western diet	Global knockout	↘	↘	↘	ND	(40)
	High-carbohydrate diet	Liver-specific knockout	↘	↘	↘	↘	(34)
	HFrD	Global knockout	↘	ND	↗	ND	(41)
		Liver-specific knockout	↘	↘	=	↗	(42)
MondoA	Standard diet	Global knockout	=	ND	ND	=	(43)
		Muscle-specific knockout	=	ND	ND	=	(44)
	High-fat diet	Muscle-specific knockout	=	ND	ND	↗	(44)
MondoA /ChREBP	High-fat diet	Administration of a compound (SBI-993) that deactivates MondoA/ChREBP signaling	↘	ND	↘	↗	(45)

“=“ means not changed. ND, not determined. The upward and downward arrows indicate an increase and decrease in the level, respectively.

### MondoA in Metabolic Organs

As another transcriptional biosensor of intracellular glucose concentration, MondoA contributes to more than 75% of glucose-induced transcription signature in HA1ER epithelial cells (52). By shuttling between mitochondria and nucleus, MondoA bridges cytoplasmic nutrient level to transcriptional adaptations. MondoA localizes to the outer mitochondrial membrane under basal conditions, and accumulates in the nucleus in response to nutrient signals such as high glucose (52, 53). In addition to nuclear accumulation, glucose triggers MondoA-MLX binding to target promoters, and activates gene expression through recruitment of histone H3 acetyltransferase as coactivators (54).

Similar to ChREBP, MondoA senses levels of G6P and F2,6-BP (52, 55) (**Figure 2**). Meanwhile, MondoA is responsive to non-glucose hexoses including allose and glucosamine (56). Intriguingly, glutamine recruits a histone deacetylase-dependent corepressor to MondoA, turning MondoA-MLX into a transcriptional repressor. Moreover, cellular acidosis drives MondoA transcriptional activity since low pH promotes the production of mitochondrial ATP, with which mitochondria-bound hexokinase generates G6P from cytoplasmic glucose (57). This finding has justified the special localization of MondoA-MLX and unraveled the mechanisms underlying the activation of MondoA by lactic acidosis. Other molecules reported to be sensed by MondoA include adenine nucleotides and other adenosine-containing molecules (58, 59). Furthermore, mTOR (mammalian target of rapamycin), another key nutrient sensor, interacts with MondoA with a suppressive effect on its transcriptional activity (60). Nonetheless, so far there is no report on fatty acids or amino acids regulating MondoA level or activity.

Different from ChREBP, MondoA is predominantly expressed in skeletal muscle and immune cells (12), and our unpublished data. MondoA-deficient mice show enhanced glycolytic rates probably because loss of MondoA in skeletal muscle increases glucose uptake (43) (**Table 1**). In response to glucose and fructose, MondoA activates transcription of thioredoxin interacting protein (TXNIP) and arrestin domain-containing 4 (ARRDC4), which inhibits glucose uptake (**Figure 3**) (56, 61). TXNIP, a dynamic sensor that modulates the energy demand of cells, plays a crucial role in the homeostasis of glucose. As a direct and glucose-responsive target of MondoA, TXNIP is upregulated when G6P level increases and concomitantly restricts glucose absorption, thus providing a negative feedback loop to prevent energy overload. Mechanisms underlying inhibition of glucose uptake regulated by TXNIP include the suppression of glucose transporter (GLUT) expression, GLUT vesicle transport and insulin signaling (44, 62–64). Moreover, MondoA enhances glycogen synthesis by activating the transcription of phosphoprotein phosphatase 1 regulatory subunit 3A (PPP1R3A), phosphoprotein phosphatase 1 regulatory subunit 3B (PPP1R3B) and genes encoding the glycogen targeting subunits of protein phosphatase 1 (PP1) for promoting glycogen synthesis (65, 66). Muscle-specific MondoA knockout decreases glycogen level in the skeletal muscle of mice (62) (**Table 1**). Hence, under physiological conditions, glucose homeostasis is maintained by the downstream effects of MondoA activation.

In addition to glucose metabolism, MondoA diverts nutrients to lipid metabolic pathways, including fatty acid thioesterification [acyl-CoA synthetase 1, 4 (ACSL1, 4)], desaturation [fatty acid desaturase 1,2 (FADS1, 2), SCD1, 5], elongation [elongation of very long chain fatty acids protein 5, 6 (ELOVL5, 6)], and triglyceride synthesis [diacylglycerol acyltransferase 1, 2 (DGAT1, 2)] (44) (**Figure 3**). Taken together, as a nutrient-regulated transcription factor, MondoA not only decreases glucose import but also diverts nutrients to storage in skeletal muscle. Although various posttranslational modifications of ChREBP have been revealed to regulate its activity in different conditions (67–69), there is so far no report on how MondoA is posttranslationally modified. Therefore, further mechanistic studies are needed to elucidate the interacting protein network of MondoA in response to nutrient level alterations.

## The Role of MondoA and ChREBP in Obesity

### ChREBP: From White to Brown and Beige Adipocytes

Obesity is the excessive accumulation of fat caused by imbalance between energy intake and consumption. It is the major risk factor for many metabolic disorders such as type 2 diabetes, fatty liver and cardiovascular diseases (70). Regarded as a crucial target for the prevention and treatment of obesity, the adipose tissue consists of white adipocytes which store energy, and brown and beige adipocytes which consume energy and produce heat (71). Inducing beige adipocytes from white adipose tissues (WAT) is known as browning, a process which improves glucose metabolism and insulin sensitivity (11). Various transcription and endocrine factors participate in this process by directly or indirectly stimulating UCP1 expression in adipose tissues, including peroxisome proliferator-activated receptor γ (PPARγ), PPARγ coactivator-1α (PGC-1α), silent information regulator type 1 (SIRT1) and FGF21 (72). The activation of brown and beige adipocytes is considered to be an attractive therapeutic strategy for obesity and its comorbidities. Brown and beige adipocytes serve as a sink for excessive nutrients by promoting energy expenditure in mitochondria (73).

ChREBP promotes lipogenesis in adipose tissues (36, 74). For high-carbohydrate diets, excessive fructose and glucose are converted to fatty acids, in which a series of enzymes including ACLY, ACC and FAS participate (75). The predominant destiny of the newly synthesized fatty acids is to become triglycerides for storage, which helps to maintain energy homeostasis (76). Adipocyte *de novo* lipogenesis is also involved in the regulation of systemic insulin sensitivity and thermogenesis, both of which play key roles in mediating metabolic adaptations (21, 77). Overexpression of a constitutively active ChREBP isoform (caChREBP) in adipose tissues leads to an increase in expression of key enzymes involved in *de novo* lipogenesis (37). Conversely, adipocytes lacking ChREBP display impaired sucrose-induced lipogenesis (36). In WAT, Glut4-mediated glucose uptake induces ChREBP expression and activates the *de novo* lipogenic pathway. In Glut4 knockout mice, ChREBP expression in adipose tissues decreases by 50%. It is noteworthy that Glut4-mediated changes in glucose flux have a stronger effect on the transcriptional expression of ChREBPβ than ChREBPα in WAT (16). ChREBP activity in adipocytes depends on ACLY, one of its transcriptional targets. In the absence of ACLY, the expression of both ChREBP and its target genes is significantly suppressed. Consequently, ACLY and ChREBP form a positive feedback loop in adipocytes to foster dietary carbohydrates uptake, fatty acid synthesis and storage of lipids (78). Moreover, specific ablation of Rictor, the essential subunit of the mechanistic target of rapamycin complex 2 (mTORC2) in mature adipocytes reduces ChREBPβ expression and downregulates *de novo* lipogenesis in WAT and brown adipose tissue (BAT) (79). In mature brown adipocytes, AKT2, which can be phosphorylated by mTORC2, is required for lipogenesis driven by ChREBP activation (21, 80).

**Figure 4:**
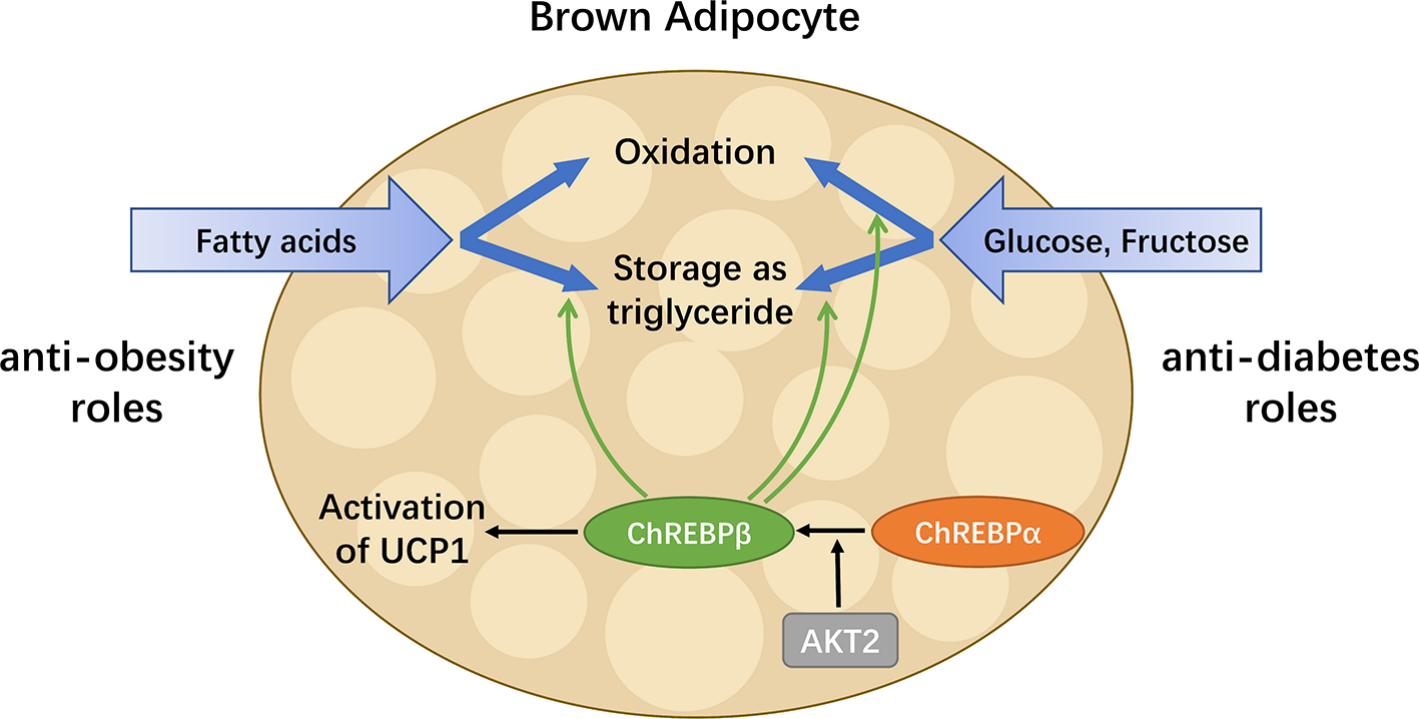
ChREBP in BAT combats metabolic diseases. BAT has enormous promise for treating metabolic diseases including obesity and diabetes, as it is capable of taking up glucose, fructose and fatty acids, as well as oxidizing or storing them afterwards. ChREBPβ, a truncated isoform of ChREBP, is most highly expressed in BAT and induced by the activation of the canonical isoform ChREBPα. In addition to regulating key enzymes involved in metabolism pathways of glucose, fructose and fatty acids, ChREBP also mediates the activation of UCP1 in brown adipocytes. Thus, in BAT, ChREBP plays both anti-obesity and anti-diabetes roles by increasing energy expenditure, reducing circulating glucose and improving insulin sensitivity.

To date, the role of ChREBP in regulating thermogenic adipocyte function has been indicated in a growing body of literatures (**Figure 4**). Reduced brown fat mass and hypothermia in response to excess energy intake are observed in ChREBP-deficient mice (32). ChREBPβ and UCP1 expression levels positively correlate in human BAT, suggesting that ChREBPβ expression might indicate brown adipocyte activity (21). Under chronic cold exposure, specific impairment of ChREBP-driven lipogenesis in BAT promotes beige adipocyte development, which is probably a compensatory response (21). Moreover, studies in adipocytes exposed to high glucose show that ChREBP is a critical mediator in triiodothyronine-induced upregulation of UCP1 expression in brown adipocytes (81). However, there is no significant binding of ChREBP protein to the UCP1 promoter, indicating that ChREBP regulates UCP1 transcription indirectly (82), which awaits further study. In mice fed a chronic high sucrose diet, expression of UCP1 in BAT is significantly increased compared with controls, which is probably due to the activated ChREBP-FGF21 axis (83). Moreover, overexpression of constitutively active ChREBP in adipocytes induces PPARγ activity and upregulates its thermogenesis-related target genes including UCP1 that promotes browning of WAT, while depletion of endogenous ChREBP in adipocytes has reciprocal effects (84). Furthermore, adenoviral overexpression of ChREBP in mice increases mRNA level of white adipose tissue UCP1 with increased plasma FGF21 level (46). After ChREBPβ is identified, it is important to consider the functional difference between the two isoforms of ChREBP. Of note, overexpression of ChREBPβ in brown adipocytes leads to impaired BAT thermogenesis and WAT browning, reflecting the role of ChREBPβ as a feedback regulator upon cold exposure (82). Moreover, given the expression of ChREBP in metabolic organs and macrophages, global gain-of-function or loss-of-function mouse models of ChREBP may not be ideal for analyzing the role of ChREBP in adipose tissues. Additional studies will be needed to develop a full picture of the specific role and mechanism of the two isoforms of ChREBP in adipocyte thermogenesis.

### MondoA: Inter-Organ Metabolic Crosstalk

MondoA is expressed predominantly in skeletal muscle which makes up ~40% of body weight and is responsible for ~80% of glucose uptake (62, 85). Therefore, as a transcriptional factor required for maintaining body homeostasis, MondoA plays a crucial part in inter-organ metabolic crosstalk.

In the context of chronic energy overload, MondoA is activated by glucose and fructose, which leads to the upregulation of TXNIP and ARRDC4, and concomitantly the impairment of glucose uptake in the skeletal muscle *via* suppression of insulin action (44, 45, 55). Additionally, the chronic activation of MondoA in skeletal muscle results in lipotoxicity, namely deleterious effects of ectopic triglyceride accumulation (44, 45, 55, 86). Therefore, MondoA activation results in myocellular insulin resistance and lipid accumulation (44), serving as an intriguing supplement to the well-known insulin resistance based on triglyceride accumulation (87). These defects, along with hyperglycemia and hyperlipidemia, contribute to obesity-induced type 2 diabetes (T2D) (88).

In obesity, pancreatic β cells adaptively produce more insulin to maintain blood glucose level, resulting in an amplified burden on β cells. Intriguingly, MondoA serves as a significant glucose-responsive transcription factor in β cells (89). Under high glucose conditions, MondoA shuttles to the nucleus to induce its targets TXNIP and ARRDC4 in β cells. TXNIP is a major factor promoting β cell apoptosis (89–93). Hence, impaired β cells might lead to progressive dysfunction of pancreas and even loss of its ability to produce and secrete insulin (94, 95).

All facts mentioned above will lead to an inter-organ vicious cycle of nutrient disposal and metabolism. The ideal solution is to limit energy intake while increasing the aerobic oxidation of fat in skeletal muscle by exercising (88). Therefore, to get out of the vicious cycle and regain the virtuous cycle, it is worthwhile to treat MondoA in skeletal muscle as a therapeutic target for obesity and insulin resistance.

## Mondo Family as a Target for Metabolic Disorders

In view of the central role of Mondo family in regulating energy homeostasis, the possibility to target MondoA or ChREBP in metabolic disorders has been explored.

As MondoA downregulates insulin sensitivity and promotes lipid storage in skeletal muscle (44, 45), it could be a promising therapeutic target for insulin resistance and lipotoxicity. For diet-induced obesity, muscle triglyceride accumulation and insulin resistance are partially relieved in muscle-specific MondoA knockout mice (44). MondoA deletion increases muscle glucose uptake and glycolytic capacity, resulting in enhanced sprint capacity (43). Moreover, SBI-477, a potent inhibitor of MondoA, alleviates muscle triglyceride levels and hepatic steatosis, thereby improving glucose tolerance in mice on a high-fat diet (45). However, the significant role of MondoA in skeletal muscle development has been recently revealed in mice (62). Therefore, in the development of MondoA as a novel therapeutic target, the timing for treatment is critical and the risk of interfering with normal myogenesis needs to be avoided.

Reducing ChREBP activity is considered as a promising target in the treatment of obesity according to studies utilizing *ob/ob* and ChREBP double knockout mice (33). Of note, ChREBP plays an important role in promoting white adipocyte browning (81). Therefore, ChREBP in brown and beige adipocytes can be regarded as a treatment option for obesity. In consideration of the well-established role of brown and beige adipocytes in counteracting obesity, we await further research in this respect.

## Summary

Mondo family transcription factors are critical for metabolic homeostasis, as they sense multiple nutrient molecules and regulate metabolic enzyme genes transcriptionally. MondoA limits glucose uptake and glycolysis mostly in skeletal muscle and immune cells, while ChREBP promotes *de novo* lipogenesis in liver and adipose tissue. In pathological states of nutrient overload, MondoA could interfere with insulin signaling, while adipose ChREBP is linked to systemic insulin sensitivity and its role extends from white to brown and beige adipose tissues. The role of ChREBP in browning of white adipocytes is especially worth further exploration. Targeting MondoA and ChREBP to counteract obesity and related diseases is an appealing strategy that requires further investigations. As the manipulation of Mondo family in different organs and tissues could yield distinct systemic metabolic consequences, future studies should be conducted using more specific and rigorous models in order to clarify the beneficial or deleterious effects of Mondo family in different contexts. Meanwhile, before the therapeutic approaches could be developed, it is noteworthy that MondoA and ChREBP could be involved in normal myogenesis and adipogenesis. Moreover, under certain circumstances, the target genes and metabolic pathways of MondoA and ChREBP are overlapping. In this regard, in the knockout phenotype of one of the two transcription factors, whether the other acts in a compensatory way requires special attention. Furthermore, increasing studies reveal the involvement of Mondo family in critical signaling pathways, which awaits mechanistic investigations to expand our understanding of the action and regulation of these transcription factors.

## Author Contributions

XT designed and revised the manuscript. HK, YL, SW, and YZ wrote the manuscript, made the figures and the table. All authors contributed to the article and approved the submitted version.

## Funding

This work was supported by grants from the 14th Undergraduate Training Program for Innovation of Shanghai Jiao Tong University School of Medicine (1420Y002), from National Natural Science Foundation of China (81672322, 81972210 and 92057117); the Shanghai Municipal Science and Technology Major Project (19JC1410200); National Key R&D Program of China (2019YFA0906100); National Key Research and Development Program of China (No. 2016YFC1304800); Shanghai Jiao Tong University School of Medicine and Innovative research team of high-level local universities in Shanghai (SSMU-ZDCX20180400); The Program for Professor of Special Appointment (Eastern Scholar) at Shanghai Institutions of Higher Learning and Construction; Plan of Laboratory Technical Team in Shanghai Universities (SYjdyx19019).

## Conflict of Interest

The authors declare that the research was conducted in the absence of any commercial or financial relationships that could be construed as a potential conflict of interest.

## References

[1] GBD 2017 Risk Factor Collaborators. Global, regional, and national comparative risk assessment of 84 behavioural, environmental and occupational, and metabolic risks or clusters of risks for 195 countries and territories, 1990-2017: a systematic analysis for the Global Burden of Disease Study 2017. Lancet (2018) 392:1923–94. 10.1016/s0140-6736(18)32225-6 PMC6227755 30496105

[2] Ludwig DS Peterson KE Gortmaker SL. Relation between consumption of sugar-sweetened drinks and childhood obesity: a prospective, observational analysis. Lancet (2001) 357:505–8. 10.1016/s0140-6736(00)04041-1 11229668

[3] Schulze MB Manson JE Ludwig DS Colditz GA Stampfer MJ Willett WC. Sugar-sweetened beverages, weight gain, and incidence of type 2 diabetes in young and middle-aged women. Jama (2004) 292:927–34. 10.1001/jama.292.8.927 15328324

[4] Sievenpiper JL de Souza RJ Mirrahimi A Yu ME Carleton AJ Beyene J. Effect of fructose on body weight in controlled feeding trials: a systematic review and meta-analysis. Ann Intern Med (2012) 156:291–304. 10.7326/0003-4819-156-4-201202210-00007 22351714

[5] Harms M Seale P. Brown and beige fat: development, function and therapeutic potential. Nat Med (2013) 19:1252–63. 10.1038/nm.3361 24100998

[6] Cederberg A Grønning LM Ahrén B Taskén K Carlsson P Enerbäck S. FOXC2 is a winged helix gene that counteracts obesity, hypertriglyceridemia, and diet-induced insulin resistance. Cell (2001) 106:563–73. 10.1016/s0092-8674(01)00474-3 11551504

[7] Kopecky J Clarke G Enerbäck S Spiegelman B Kozak LP. Expression of the mitochondrial uncoupling protein gene from the aP2 gene promoter prevents genetic obesity. J Clin Invest (1995) 96:2914–23. 10.1172/jci118363 PMC186003 8675663

[8] Seale P Conroe HM Estall J Kajimura S Frontini A Ishibashi J. Prdm16 determines the thermogenic program of subcutaneous white adipose tissue in mice. J Clin Invest (2011) 121:96–105. 10.1172/jci44271 21123942 PMC3007155

[9] Ortega-Molina A Efeyan A Lopez-Guadamillas E Muñoz-Martin M Gómez-López G Cañamero M. Pten positively regulates brown adipose function, energy expenditure, and longevity. Cell Metab (2012) 15:382–94. 10.1016/j.cmet.2012.02.001 22405073

[10] Vegiopoulos A Müller-Decker K Strzoda D Schmitt I Chichelnitskiy E Ostertag A. Cyclooxygenase-2 controls energy homeostasis in mice by *de novo* recruitment of brown adipocytes. Science (2010) 328:1158–61. 10.1126/science.1186034 20448152

[11] Lee P Smith S Linderman J Courville AB Brychta RJ Dieckmann W. Temperature-acclimated brown adipose tissue modulates insulin sensitivity in humans. Diabetes (2014) 63:3686–98. 10.2337/db14-0513 PMC4207391 24954193

[12] Billin AN Eilers AL Coulter KL Logan JS Ayer DE. MondoA, a novel basic helix-loop-helix-leucine zipper transcriptional activator that constitutes a positive branch of a max-like network. Mol Cell Biol (2000) 20:8845–54. 10.1128/mcb.20.23.8845-8854.2000 PMC86535 11073985

[13] Yamashita H Takenoshita M Sakurai M Bruick RK Henzel WJ Shillinglaw W. A glucose-responsive transcription factor that regulates carbohydrate metabolism in the liver. Proc Natl Acad Sci USA (2001) 98:9116–21. 10.1073/pnas.161284298 PMC55382 11470916

[14] Stoeckman AK Ma L Towle HC. Mlx is the functional heteromeric partner of the carbohydrate response element-binding protein in glucose regulation of lipogenic enzyme genes. J Biol Chem (2004) 279:15662–9. 10.1074/jbc.M311301200 14742444

[15] Li MV Chang B Imamura M Poungvarin N Chan L. Glucose-dependent transcriptional regulation by an evolutionarily conserved glucose-sensing module. Diabetes (2006) 55:1179–89. 10.2337/db05-0822 16644671

[16] Herman MA Peroni OD Villoria J Schön MR Abumrad NA Blüher M. A novel ChREBP isoform in adipose tissue regulates systemic glucose metabolism. Nature (2012) 484:333–8. 10.1038/nature10986 PMC3341994 22466288

[17] Ge Q Nakagawa T Wynn RM Chook YM Miller BC Uyeda K. Importin-alpha protein binding to a nuclear localization signal of carbohydrate response element-binding protein (ChREBP). J Biol Chem (2011) 286:28119–27. 10.1074/jbc.M111.237016 PMC3151057 21665952

[18] Eilers AL Sundwall E Lin M Sullivan AA Ayer DE. A novel heterodimerization domain, CRM1, and 14-3-3 control subcellular localization of the MondoA-Mlx heterocomplex. Mol Cell Biol (2002) 22:8514–26. 10.1128/mcb.22.24.8514-8526.2002 PMC139889 12446771

[19] Sakiyama H Wynn RM Lee WR Fukasawa M Mizuguchi H Gardner KH. Regulation of nuclear import/export of carbohydrate response element-binding protein (ChREBP): interaction of an alpha-helix of ChREBP with the 14-3-3 proteins and regulation by phosphorylation. J Biol Chem (2008) 283:24899–908. 10.1074/jbc.M804308200 PMC3259841 18606808

[20] Li MV Chen W Poungvarin N Imamura M Chan L. Glucose-mediated transactivation of carbohydrate response element-binding protein requires cooperative actions from Mondo conserved regions and essential trans-acting factor 14-3-3. Mol Endocrinol (2008) 22:1658–72. 10.1210/me.2007-0560 PMC2453601 18436566

[21] Sanchez-Gurmaches J Tang Y Jespersen NZ Wallace M Martinez Calejman C Gujja S. Brown Fat AKT2 Is a Cold-Induced Kinase that Stimulates ChREBP-Mediated De Novo Lipogenesis to Optimize Fuel Storage and Thermogenesis. Cell Metab (2018) 27:195–209. 10.1016/j.cmet.2017.10.008 29153407 PMC5762420

[22] Dentin R Tomas-Cobos L Foufelle F Leopold J Girard J Postic C. Glucose 6-phosphate, rather than xylulose 5-phosphate, is required for the activation of ChREBP in response to glucose in the liver. J Hepatol (2012) 56:199–209. 10.1016/j.jhep.2011.07.019 21835137

[23] McFerrin LG Atchley WR. A novel N-terminal domain may dictate the glucose response of Mondo proteins. PloS One (2012) 7:e34803. 10.1371/journal.pone.0034803 22506051 PMC3323566

[24] Kabashima T Kawaguchi T Wadzinski BE Uyeda K. Xylulose 5-phosphate mediates glucose-induced lipogenesis by xylulose 5-phosphate-activated protein phosphatase in rat liver. Proc Natl Acad Sci USA (2003) 100:5107–12. 10.1073/pnas.0730817100 PMC154306 12684532

[25] Arden C Tudhope SJ Petrie JL Al-Oanzi ZH Cullen KS Lange AJ. Fructose 2,6-bisphosphate is essential for glucose-regulated gene transcription of glucose-6-phosphatase and other ChREBP target genes in hepatocytes. Biochem J (2012) 443:111–23. 10.1042/bj20111280 22214556

[26] Sato S Jung H Nakagawa T Pawlosky R Takeshima T Lee WR. Metabolite Regulation of Nuclear Localization of Carbohydrate-response Element-binding Protein (ChREBP): ROLE OF AMP AS AN ALLOSTERIC INHIBITOR. J Biol Chem (2016) 291:10515–27. 10.1074/jbc.M115.708982 PMC4865902 26984404

[27] Nakagawa T Ge Q Pawlosky R Wynn RM Veech RL Uyeda K. Metabolite regulation of nucleo-cytosolic trafficking of carbohydrate response element-binding protein (ChREBP): role of ketone bodies. J Biol Chem (2013) 288:28358–67. 10.1074/jbc.M113.498550 PMC3784753 23918932

[28] Postic C Dentin R Denechaud P-D Girard J. ChREBP, a transcriptional regulator of glucose and lipid metabolism. Annu Rev Nutr (2007) 27:179–92. 10.1146/annurev.nutr.27.061406.093618 17428181

[29] Ishii S Iizuka K Miller BC Uyeda K. Carbohydrate response element binding protein directly promotes lipogenic enzyme gene transcription. Proc Natl Acad Sci USA (2004) 101:15597–602. 10.1073/pnas.0405238101 PMC524841 15496471

[30] Ma L Robinson LN Towle HC. ChREBP*Mlx is the principal mediator of glucose-induced gene expression in the liver. J Biol Chem (2006) 281:28721–30. 10.1074/jbc.M601576200 16885160

[31] Niwa H Iizuka K Kato T Wu W Tsuchida H Takao K. ChREBP Rather Than SHP Regulates Hepatic VLDL Secretion. Nutrients (2018) 10:321. 10.3390/nu10030321 PMC5872739 29518948

[32] Iizuka K Bruick RK Liang G Horton JD Uyeda K. Deficiency of carbohydrate response element-binding protein (ChREBP) reduces lipogenesis as well as glycolysis. Proc Natl Acad Sci USA (2004) 101:7281–6. 10.1073/pnas.0401516101 PMC409910 15118080

[33] Iizuka K Miller B Uyeda K. Deficiency of carbohydrate-activated transcription factor ChREBP prevents obesity and improves plasma glucose control in leptin-deficient (ob/ob) mice. Am J Physiol Endocrinol Metab (2006) 291:E358–64. 10.1152/ajpendo.00027.2006 16705063

[34] Jois T Chen W Howard V Harvey R Youngs K Thalmann C. Deletion of hepatic carbohydrate response element binding protein (ChREBP) impairs glucose homeostasis and hepatic insulin sensitivity in mice. Mol Metab (2017) 6:1381–94. 10.1016/j.molmet.2017.07.006 PMC5681238 29107286

[35] Benhamed F Denechaud PD Lemoine M Robichon C Moldes M Bertrand-Michel J. The lipogenic transcription factor ChREBP dissociates hepatic steatosis from insulin resistance in mice and humans. J Clin Invest (2012) 122:2176–94. 10.1172/JCI41636 PMC3366390 22546860

[36] Vijayakumar A Aryal P Wen J Syed I Vazirani RP Moraes-Vieira PM. Absence of Carbohydrate Response Element Binding Protein in Adipocytes Causes Systemic Insulin Resistance and Impairs Glucose Transport. Cell Rep (2017) 21:1021–35. 10.1016/j.celrep.2017.09.091 PMC5771491 29069585

[37] Nuotio-Antar AM Poungvarin N Li M Schupp M Mohammad M Gerard S. FABP4-Cre Mediated Expression of Constitutively Active ChREBP Protects Against Obesity, Fatty Liver, and Insulin Resistance. Endocrinology (2015) 156:4020–32. 10.1210/en.2015-1210 PMC4606753 26248218

[38] Poungvarin N Lee JK Yechoor VK Li MV Assavapokee T Suksaranjit P. Carbohydrate response element-binding protein (ChREBP) plays a pivotal role in beta cell glucotoxicity. Diabetologia (2012) 55:1783–96. 10.1007/s00125-012-2506-4 PMC4010252 22382520

[39] Dentin R Benhamed F Hainault I Fauveau V Foufelle F Dyck JR. Liver-specific inhibition of ChREBP improves hepatic steatosis and insulin resistance in ob/ob mice. Diabetes (2006) 55:2159–70. 10.2337/db06-0200 16873678

[40] Wu W Tsuchida H Kato T Niwa H Horikawa Y Takeda J. Fat and carbohydrate in western diet contribute differently to hepatic lipid accumulation. Biochem Biophys Res Commun (2015) 461:681–6. 10.1016/j.bbrc.2015.04.092 25931000

[41] Zhang D Tong X VanDommelen K Gupta N Stamper K Brady GF. Lipogenic transcription factor ChREBP mediates fructose-induced metabolic adaptations to prevent hepatotoxicity. J Clin Invest (2017) 127:2855–67. 10.1172/jci89934 PMC5490767 28628040

[42] Kim M Astapova II Flier SN Hannou SA Doridot L Sargsyan A. Intestinal, but not hepatic, ChREBP is required for fructose tolerance. JCI Insight (2017) 2:e96703. 10.1172/jci.insight.96703 PMC5752301 29263303

[43] Imamura M Chang BH Kohjima M Li M Hwang B Taegtmeyer H. MondoA deficiency enhances sprint performance in mice. Biochem J (2014) 464:35–48. 10.1042/bj20140530 25145386 PMC4410994

[44] Ahn B Wan S Jaiswal N Vega RB Ayer DE Titchenell PM. MondoA drives muscle lipid accumulation and insulin resistance. JCI Insight (2019) 5:e129119. 10.1172/jci.insight.129119 PMC6693825 31287806

[45] Ahn B Soundarapandian MM Sessions H Peddibhotla S Roth GP Li JL. MondoA coordinately regulates skeletal myocyte lipid homeostasis and insulin signaling. J Clin Invest (2016) 126:3567–79. 10.1172/jci87382 PMC5004938 27500491

[46] Iizuka K Takao K Kato T Horikawa Y Takeda J. ChREBP Reciprocally Regulates Liver and Plasma Triacylglycerol Levels in Different Manners. Nutrients (2018) 10:1699. 10.3390/nu10111699 PMC6266805 30405056

[47] Linden AG Li S Choi HY Fang F Fukasawa M Uyeda K. Interplay between ChREBP and SREBP-1c coordinates postprandial glycolysis and lipogenesis in livers of mice. J Lipid Res (2018) 59:475–87. 10.1194/jlr.M081836 PMC5832931 29335275

[48] White PJ McGarrah RW Grimsrud PA Tso SC Yang WH Haldeman JM. and Lipid Metabolism *via* Regulation of ATP-Citrate Lyase. Cell Metab (2018) 27:1281–93.e7. 10.1016/j.cmet.2018.04.015 29779826 PMC5990471

[49] Iizuka K Takeda J Horikawa Y. Glucose induces FGF21 mRNA expression through ChREBP activation in rat hepatocytes. FEBS Lett (2009) 583:2882–6. 10.1016/j.febslet.2009.07.053 19660458

[50] von Holstein-Rathlou S BonDurant LD Peltekian L Naber MC Yin TC Claflin KE. FGF21 Mediates Endocrine Control of Simple Sugar Intake and Sweet Taste Preference by the Liver. Cell Metab (2016) 23:335–43. 10.1016/j.cmet.2015.12.003 PMC4756759 26724858

[51] Fisher FM Kim M Doridot L Cunniff JC Parker TS Levine DM. A critical role for ChREBP-mediated FGF21 secretion in hepatic fructose metabolism. Mol Metab (2017) 6:14–21. 10.1016/j.molmet.2016.11.008 28123933 PMC5220398

[52] Stoltzman CA Peterson CW Breen KT Muoio DM Billin AN Ayer DE. Glucose sensing by MondoA : Mlx complexes: a role for hexokinases and direct regulation of thioredoxin-interacting protein expression. Proc Natl Acad Sci USA (2008) 105:6912–7. 10.1073/pnas.0712199105 PMC2383952 18458340

[53] Sans CL Satterwhite DJ Stoltzman CA Breen KT Ayer DE. MondoA-Mlx heterodimers are candidate sensors of cellular energy status: mitochondrial localization and direct regulation of glycolysis. Mol Cell Biol (2006) 26:4863–71. 10.1128/mcb.00657-05 PMC1489152 16782875

[54] Peterson CW Stoltzman CA Sighinolfi MP Han KS Ayer DE. Glucose controls nuclear accumulation, promoter binding, and transcriptional activity of the MondoA-Mlx heterodimer. Mol Cell Biol (2010) 30:2887–95. 10.1128/mcb.01613-09 PMC2876681 20385767

[55] Petrie JL Al-Oanzi ZH Arden C Tudhope SJ Mann J Kieswich J. Glucose induces protein targeting to glycogen in hepatocytes by fructose 2,6-bisphosphate-mediated recruitment of MondoA to the promoter. Mol Cell Biol (2013) 33:725–38. 10.1128/mcb.01576-12 PMC3571345 23207906

[56] Stoltzman CA Kaadige MR Peterson CW Ayer DE. MondoA senses non-glucose sugars: regulation of thioredoxin-interacting protein (TXNIP) and the hexose transport curb. J Biol Chem (2011) 286:38027–34. 10.1074/jbc.M111.275503 PMC3207397 21908621

[57] Wilde BR Ye Z Lim TY Ayer DE. Cellular acidosis triggers human MondoA transcriptional activity by driving mitochondrial ATP production. Elife (2019) 8:e40199. 10.7554/eLife.40199 30717828 PMC6363388

[58] Yu FX Goh SR Dai RP Luo Y. Adenosine-containing molecules amplify glucose signaling and enhance txnip expression. Mol Endocrinol (2009) 23:932–42. 10.1210/me.2008-0383 PMC5419282 19246513

[59] Han KS Ayer DE. MondoA senses adenine nucleotides: transcriptional induction of thioredoxin-interacting protein. Biochem J (2013) 453:209–18. 10.1042/bj20121126 PMC4435952 23631812

[60] Kaadige MR Yang J Wilde BR Ayer DE. MondoA-Mlx transcriptional activity is limited by mTOR-MondoA interaction. Mol Cell Biol (2015) 35:101–10. 10.1128/mcb.00636-14 PMC4295369 25332233

[61] Parikh H Carlsson E Chutkow WA Johansson LE Storgaard H Poulsen P. TXNIP regulates peripheral glucose metabolism in humans. PloS Med (2007) 4:e158. 10.1371/journal.pmed.0040158 17472435 PMC1858708

[62] Ran H Lu Y Zhang Q Hu Q Zhao J Wang K. MondoA Is Required for Normal Myogenesis and Regulation of the Skeletal Muscle Glycogen Content in Mice. Diabetes Metab J (2020). 10.4093/dmj.2019.0212 PMC8497918 34610727

[63] Mandala A Das N Bhattacharjee S Mukherjee B Mukhopadhyay S Roy SS. Thioredoxin interacting protein mediates lipid-induced impairment of glucose uptake in skeletal muscle. Biochem Biophys Res Commun (2016) 479:933–9. 10.1016/j.bbrc.2016.09.168 27702549

[64] Waldhart AN Dykstra H Peck AS Boguslawski EA Madaj ZB Wen J. Phosphorylation of TXNIP by AKT Mediates Acute Influx of Glucose in Response to Insulin. Cell Rep (2017) 19:2005–13. 10.1016/j.celrep.2017.05.041 PMC5603216 28591573

[65] Delibegovic M Armstrong CG Dobbie L Watt PW Smith AJH Cohen PTW. Disruption of the striated muscle glycogen targeting subunit PPP1R3A of protein phosphatase 1 leads to increased weight gain, fat deposition, and development of insulin resistance. Diabetes (2003) 52:596–604. 10.2337/diabetes.52.3.596 12606498

[66] Montori-Grau M Guitart M Lerin C Andreu AL Newgard CB García-Martínez C. Expression and glycogenic effect of glycogen-targeting protein phosphatase 1 regulatory subunit GL in cultured human muscle. Biochem J (2007) 405:107–13. 10.1042/BJ20061572 PMC1925244 17555403

[67] Kawaguchi T Takenoshita M Kabashima T Uyeda K. Glucose and cAMP regulate the L-type pyruvate kinase gene by phosphorylation/dephosphorylation of the carbohydrate response element binding protein. Proc Natl Acad Sci USA (2001) 98:13710–5. 10.1073/pnas.231370798 PMC61106 11698644

[68] Bricambert J Miranda J Benhamed F Girard J Postic C Dentin R. Salt-inducible kinase 2 links transcriptional coactivator p300 phosphorylation to the prevention of ChREBP-dependent hepatic steatosis in mice. J Clin Invest (2010) 120:4316–31. 10.1172/jci41624 PMC2993582 21084751

[69] Guinez C Filhoulaud G Rayah-Benhamed F Marmier S Dubuquoy C Dentin R. O-GlcNAcylation increases ChREBP protein content and transcriptional activity in the liver. Diabetes (2011) 60:1399–413. 10.2337/db10-0452 PMC3292313 21471514

[70] Carobbio S Pellegrinelli V Vidal-Puig A. Adipose Tissue Function and Expandability as Determinants of Lipotoxicity and the Metabolic Syndrome. Adv Exp Med Biol (2017) 960:161–96. 10.1007/978-3-319-48382-5_7 28585199

[71] Lynes MD Tseng Y-H. Deciphering adipose tissue heterogeneity. Ann N Y Acad Sci (2018) 1411:5–20 10.1111/nyas.13398 28763833 PMC5788721

[72] Montanari T Pošćić N Colitti M. Factors involved in white-to-brown adipose tissue conversion and in thermogenesis: a review. Obes Rev (2017) 18:495–513. 10.1111/obr.12520 28187240

[73] Ricquier D. UCP1, the mitochondrial uncoupling protein of brown adipocyte: A personal contribution and a historical perspective. Biochimie (2017) 134:3–8. 10.1016/j.biochi.2016.10.018 27916641

[74] Song Z Xiaoli AM Yang F. Regulation and Metabolic Significance of Lipogenesis in Adipose Tissues. Nutrients (2018) 10:1383. 10.3390/nu10101383 PMC6213738 30274245

[75] Ameer F Scandiuzzi L Hasnain S Kalbacher H Zaidi N. De novo lipogenesis in health and disease. Metabolism (2014) 63:895–902. 10.1016/j.metabol.2014.04.003 24814684

[76] Lodhi IJ Wei X Semenkovich CF. Lipoexpediency: *de novo* lipogenesis as a metabolic signal transmitter. Trends Endocrinol Metab (2011) 22:1–8. 10.1016/j.tem.2010.09.002 20889351 PMC3011046

[77] Smith U Kahn BB. Adipose tissue regulates insulin sensitivity: role of adipogenesis, *de novo* lipogenesis and novel lipids. J Intern Med (2016) 280:465–75. 10.1111/joim.12540 PMC5218584 27699898

[78] Fernandez S Viola JM Torres A Wallace M Trefely S Zhao S. Adipocyte ACLY Facilitates Dietary Carbohydrate Handling to Maintain Metabolic Homeostasis in Females. Cell Rep (2019) 27:2772–84.e6. 10.1016/j.celrep.2019.04.112 31141698 PMC6608748

[79] Tang Y Wallace M Sanchez-Gurmaches J Hsiao WY Li H Lee PL. Adipose tissue mTORC2 regulates ChREBP-driven *de novo* lipogenesis and hepatic glucose metabolism. Nat Commun (2016) 7:11365. 10.1038/ncomms11365 27098609 PMC4844681

[80] Sanchez-Gurmaches J Martinez Calejman C Jung SM Li H Guertin DA. Brown fat organogenesis and maintenance requires AKT1 and AKT2. Mol Metab (2019) 23:60–74. 10.1016/j.molmet.2019.02.004 30833219 PMC6480051

[81] Katz LS Xu S Ge K Scott DK Gershengorn MC. T3 and Glucose Coordinately Stimulate ChREBP-Mediated Ucp1 Expression in Brown Adipocytes From Male Mice. Endocrinology (2018) 159:557–69. 10.1210/en.2017-00579 PMC5761585 29077876

[82] Wei C Ma X Su K Qi S Zhu Y Lin J. ChREBP-β regulates thermogenesis in brown adipose tissue. J Endocrinol (2020) 245:343–56. 10.1530/JOE-19-0498 32208359

[83] Maekawa R Seino Y Ogata H Murase M Iida A Hosokawa K. Chronic high-sucrose diet increases fibroblast growth factor 21 production and energy expenditure in mice. J Nutr Biochem (2017) 49:71–9. 10.1016/j.jnutbio.2017.07.010 28886439

[84] Witte N Muenzner M Rietscher J Knauer M Heidenreich S Nuotio-Antar AM. The Glucose Sensor ChREBP Links De Novo Lipogenesis to PPARgamma Activity and Adipocyte Differentiation. Endocrinology (2015) 156:4008–19. 10.1210/EN.2015-1209 26181104

[85] DeFronzo RA Gunnarsson R Björkman O Olsson M Wahren J. Effects of insulin on peripheral and splanchnic glucose metabolism in noninsulin-dependent (type II) diabetes mellitus. J Clin Invest (1985) 76:149–55. 10.1172/JCI111938 PMC423730 3894418

[86] Sartor F Jackson MJ Squillace C Shepherd A Moore JP Ayer DE. Adaptive metabolic response to 4 weeks of sugar-sweetened beverage consumption in healthy, lightly active individuals and chronic high glucose availability in primary human myotubes. Eur J Nutr (2013) 52:937–48. 10.1007/s00394-012-0401-x PMC4447301 22733000

[87] Pan DA Lillioja S Kriketos AD Milner MR Baur LA Bogardus C. Skeletal muscle triglyceride levels are inversely related to insulin action. Diabetes (1997) 46:983–8. 10.2337/diab.46.6.983 9166669

[88] Simonson DC Halperin F Foster K Vernon A Goldfine AB. Clinical and Patient-Centered Outcomes in Obese Patients With Type 2 Diabetes 3 Years After Randomization to Roux-en-Y Gastric Bypass Surgery Versus Intensive Lifestyle Management: The SLIMM-T2D Study. Diabetes Care (2018) 41:670–9. 10.2337/dc17-0487 PMC5860843 29432125

[89] Richards P Rachdi L Oshima M Marchetti P Bugliani M Armanet M. MondoA Is an Essential Glucose-Responsive Transcription Factor in Human Pancreatic β-Cells. Diabetes (2018) 67:461–72. 10.2337/db17-0595 29282201

[90] Thielen L Shalev A. Diabetes pathogenic mechanisms and potential new therapies based upon a novel target called TXNIP. Curr Opin Endocrinol Diabetes Obes (2018) 25:75–80. 10.1097/MED.0000000000000391 29356688 PMC5831522

[91] Lerner AG Upton J-P Praveen PVK Ghosh R Nakagawa Y Igbaria A. IRE1α induces thioredoxin-interacting protein to activate the NLRP3 inflammasome and promote programmed cell death under irremediable ER stress. Cell Metab (2012) 16:250–64. 10.1016/j.cmet.2012.07.007 PMC4014071 22883233

[92] Chen J Hui ST Couto FM Mungrue IN Davis DB Attie AD. Thioredoxin-interacting protein deficiency induces Akt/Bcl-xL signaling and pancreatic beta-cell mass and protects against diabetes. FASEB J: Off Publ Fed Am Societies Exp Biol (2008) 22:3581–94. 10.1096/fj.08-111690 PMC2537437 18552236

[93] Saxena G Chen J Shalev A. Intracellular shuttling and mitochondrial function of thioredoxin-interacting protein. J Biol Chem (2010) 285:3997–4005. 10.1074/jbc.M109.034421 19959470 PMC2823541

[94] Boland BB Rhodes CJ Grimsby JS. The dynamic plasticity of insulin production in β-cells. Mol Metab (2017) 6:958–73. 10.1016/j.molmet.2017.04.010 PMC5605729 28951821

[95] Robertson RP. Chronic oxidative stress as a central mechanism for glucose toxicity in pancreatic islet beta cells in diabetes. J Biol Chem (2004) 279:42351–4. 10.1074/jbc.R400019200 15258147

